# Damage Control Interventional Radiology: The bridge between non-operative management and damage control surgery

**DOI:** 10.1186/s42155-024-00485-z

**Published:** 2024-10-03

**Authors:** Velio Ascenti, Anna Maria Ierardi, Maryam Alfa-Wali, Carolina Lanza, Elika Kashef

**Affiliations:** 1 Postgraduate School of Radiology, University of Milan, Milan, IT Italy; 2 Radiology Department, Fondazione IRCCS Ca’ Granda Ospedale Maggiore Policlinico, Milan, IT Italy; 3 Major Trauma Centre, Imperial College Healthcare NHS Trust, London, UK; 4 Department of Imaging, Imperial College Healthcare NHS Trust, London, W2 1NY UK

**Keywords:** Interventional radiology, Damage control interventional radiology, Damage control surgery, Trauma

## Abstract

Traumatic injuries continue to be on the rise globally and with it, the role interventional radiology (IR) has also expanded in managing this patient cohort. The role of damage control surgery (DCS) has been well established in the trauma management pathway, however it is only recently that Damage Control IR (DCIR) has become increasingly utilized in managing the extremis trauma and emergency patient.

Visceral artery embolizations (both temporary and permanent), temporary balloon occlusions including Resuscitative Endovascular Balloon Occlusion of the Aorta (REBOA) in iliac arteries and aorta respectively are amongst the treatment options now available for the trauma (and non-traumatic bleeding) patient.

We review the literature for the role of DCS and utilization of IR in trauma, outcomes and the paradigm shift towards minimally invasive techniques. The focus of this paper is to highlight the importance of multi-disciplinary working and having established pathways to ensure timely treatment of trauma patients as well as careful patient selection.

We show that outcomes are best when both surgical and IR are involved in patient care from the outset and that DCIR should not be defined as Non-Operative Management (NOM) as it currently is categorized as.

## Introduction

Over the last 20 years there has been a continuing paradigm shift from open surgery towards more minimally invasive interventions to manage the surgical patient. Interventional Radiology (IR) has spearheaded this and is now considered first line treatment for many vascular and non vascular emergencies including being an integral part of the C- Circulation of the ABCD management of the severely injured or emergency patient.

The management of severely injured patients is divided into the primary, secondary, and tertiary surveys. The primary survey involves a rapid evaluation of the patient, resuscitation and initiation of life-saving treatment by a trauma team member. This is the ‘ABCDE’ (airway, breathing, circulation, disability, and environment) assessment of trauma. Imaging is requested as part of the primary survey while the patient is assessed. Imaging should not interfere with the flow of the primary investigation or definitive management, but only where it has an immediate impact on the initial problems of the patient [1].

Interventional radiology techniques have become part of the integral care of the trauma patient, allowing for the operator to masterfully and promptly manage the patient in extremis. In trauma, the phrase, “close the hose” is used to capture the importance of stopping the bleeding to ensure the patient’s haemodynamic status is restored as soon as possible. According to the latest guidelines of the World Society of Emergency Surgery (WSES), hemodynamic instability is defined when the patient has a systolic blood pressure < 90 mmHg, or > 90 mmHg but requiring bolus infusions/transfusions and/or vasopressor drugs and/or, base excess greater than 5 mmol/L on admission gas analysis or transfusion requirement of 4 units of packed red blood cells within the first 8 h [2]. The term unstable refers to a physiologically compromised patient requiring urgent resuscitation. Although the authors acknowledge the term unstable can be misleading, for the purpose of using a common language, in this paper the term unstable patient refers to the severely physiologically compromised patient.

The concept of DCS was first developed by Stone *et al*. in 1983 with the aim to minimize mortality in bleeding patients with coagulopathy using the technique of a ‘truncated laparotomy’. The resulted in a decrease in mortality from 98% using traditional principles of conventional laparotomy to 35% with a truncated one [3].

Definitive surgery intervention (DSI) and DCS represent the two alternatives in patients who require surgery for severe injuries depending on their haemodynamic status. A haemodynamically normal patient allows for definitive surgical intervention during the initial operation. The principles of DCS consists of an operation of a short duration to rapidly control bleeding, a substantial air leak in the chest and/or gross contamination to avoid the development the so-called “lethal or trauma triad” (acidosis, hypothermia, and coagulopathy) [4, 5]. Computed tomography (CT) is a fast, safe, crucial and effective method widely used in most trauma center for the diagnosis and the treatment of bleeding, but their use in haemodynamically unstable patients remains controversial [6]. In this cohort, patients often are taken straight to the operating theater for DCS. A novel expansion of DCS is nowadays represented by the DCIR. Differently from convention emergency interventional radiology (CEIR) focused on haemodynamically stable patients, DCIR works on haemodynamically unstable patients. The novelty of DCIR lies in its ability to provide a minimally-invasive but time-concious methods of embolization in order to control the bleeding and restore stability [6]. The aim of this review is to highlight when and why DCIR could be preferred to DCS.

### Damage control surgery

The primary goal of DCS is to control bleeding and contamination, with a focus on restoring normal physiology and not anatomy in an unstable severely injured patient.

To control bleeding in the abdomen, initial four quadrant abdominal packing with adjuvant procedures such as ligation of blood vessels, cross-clamping of the aorta, or balloon catheter tamponade are used [7]. Control of contamination is the next stage of DCS. The entire length of the gastrointestinal tract (from the oesophagus to the rectum)is examined and quick closure of the perforated viscus with suturing, ligation, or stapling is performed to prevent additional contamination. Once the patient is hemodynamically stable but certain aspects of the lethal triad remain abnormal, a temporary abdominal closure is performed, in order for the patient to be resuscitated and second-stage definitive surgery planned if appropriate. Rewarming the patient and treating their acidosis and coagulopathy are the primary goals. It is critical to treat hypothermia because, once the body temperature returns to normal, coagulopathy and acidosis can be treated and maintained [8].

Definitive surgery is planned 36-48 h after DCS for reconstruction and restoration of anatomy [7].

### Damage control interventional radiology

Interventional radiology plays a crucial role in the management of emergency and trauma patients (both blunt and penetrating injuries) and has allowed minimally invasive treatment of patients by careful case selections [9] and multidisciplinary team planning involving surgeons and anesthesiologists. In the damage control context, three different interventional techniques are used to control bleeding: temporary balloon arterial occlusion, embolization to occlude arteries (which can be either temporary or permanent), and stent grafting to repair injured vessels restoring normal flow [10]. The golden rule of DCIR is to embolize vessels as selectively as time allows.

The objective of DCIR is to confine the ongoing hemorrhage as quickly as feasible in order to maintain or restore normal hemodynamics, rather than to conduct a distal, time consuming, embolization. In this view, all procedures from catheterization to the final visual confirmation of embolization should be completed within a 10-min window for each targeted vessel [6].

For unstable patients, aggressive non-selective embolization (NSE) is preferred despite the possibility of losing vital organs.

The introduction of 24-h, 7-day IR service and advanced IR /Hybrid suite availability are imperative in offering high standard of care for the trauma patient and this is indeed part of the pre-request for many trauma centers when externally reviewed.

Although in most cases IR must be available within 60 min, the goal should be to reduce the IR availability to less than 30 min in emergency setting, to perform DCIR as quickly as feasible and improve patient outcomes [11].

### The position of DCIR in the trauma treatment algorithm?

According to the WSES guidelines, in the context of acute traumatic pathology, IR is classified as ‘non-operative management (NOM)’, and is reserved for cases in which the patient is hemodynamically stable. However, over the years, techniques, equipment and experience have evolved, and there has been an increasing need for the treatment paradigm to include endovascular experts, even for physiologically compromised trauma patients. The benefit of IR measures beyond the minimal access are the difficult-to-reach surgical sites such as the pelvis or retroperitoneum.

It is challenging to obtain high-quality data comparing the efficacy of surgery versus interventional techniques, primarily due to the inherent limitations of such comparisons. However, the increasing importance of the role of IR in emergencies is reflected by the statements of radiological and surgical societies in Europe and United States recommendations, as mentioned above, suggest that the IR team should be ready within 30-60 min from the decision to perform an angiography [12, 13]. Modern trend is to shorten that time to 30 min, like the recent document of the Royal College of Radiology reports [14].

Endovascular arterial embolization constitutes the cornerstone of DCIR. Over the past three decades, the accumulated knowledge and experience have enabled a discernment of those patients most likely to gain benefits from vascular embolization procedures in emergency settings [15].

Examples of the role of DCIR include pelvic, hepatic and splenic artery embolization, balloon tamponade in peri-partum haemorrhage and Resuscitative Endovascular Ballon Occlusion of the Aorta (REBOA).

#### Pelvic trauma

In pelvic trauma, arterial bleed is often from the branches of internal iliac artery [16]. Surgical and endovascular management are not interchangeable but complementary and deal with two different but equally important aspects of the same pathology: pre-peritoneal pelvic packing (PPP) is more effective in bleeding of venous origin as these are not amenable embolization, while endovascular embolization is more effective in bleeding of arterial origin. However, it seems that the order in which these techniques are used varies greatly depending on the local expertise, experience and the patient’s clinical status [16].

Nevertheless, whenever feasible, selective targeted embolization is preferable; if the patient has deteriorating vital parameters, rapid embolization of the entire anterior or posterior division branch of the internal iliac artery (or in rare circumstances, the internal iliac artery itself) is preferable to a time-consuming super-selective embolization of the single vessel responsible for the bleeding.

Patients in the latter group, are often obtuned or intubated therefore informed consent is not possible. In this cohort of patients, it is important to obtain careful documentation of why the decision was made to perform non-target embolization, after the procedure a candid and transparent discussion with the patient is crucial and it is responsibility of the interventional radiologist. Importantly, the increased risk of ischemia by non-selective embolization is mitigated by the rich collateral network present in this anatomical region [17]. A recent meta-analysis showed no significant difference in mortality between PPP and embolization, with 27% of patients treated with PPP requiring subsequent embolization for inadequate hemorrhage control [18]. Though, a comparison between modalities is difficult to achieve due to bias and heterogeneity of both hospitals and centers.

Reports have suggested a protocol consisting of sequentially performing, tri-modal (external fixation, PPP, and embolization), therapy of the hemodynamically unstable pelvic trauma patient ( to be the best single independent predictive factor for reducing mortality [16, 19, 20].

#### Hepatic trauma

NOM, to which endovascular embolization belongs, in hepatic trauma is effective in 50 to 85% of cases [2]. The necessary condition for NOM is hemodynamic stability according to WSES [2]. The advent of concurrent trauma hybrid resuscitation with medical and surgical management and IR embolization might enable patients to receive the appropriate treatment as soon as possible, which may be either surgical, endovascular, or a combination treatment [9, 21]. In fact, IR could be crucial also in the management of patients with hemodynamic deterioration, especially for transient responders.

Liver trauma surgery aims to control bleeding by packing the liver in addition to the use of haemostatic measures and agents if available. This is effective in controlling most venous bleeding, as it pushes the liver back into its normal anatomical position, minimizing ongoing blood loss. Nonetheless, arterial injury may still cause ongoing bleeding. In such cases, embolization may be necessary to prevent further hemorrhage. Surgical packing may not manage the inner organ arterial injury and vascular transections; in these scenarios, interventional radiology can easily work on the bleeding arterial branches for effective embolization and therefore haemorrhage control.

It is therefore crucial to determine which patients would benefit from immediate surgical intervention or angiographic investigation. This is particularly important for those who are partially responders and unstable, where time is a critical factor [9, 22]. As an analogy, if the liver can be likened to a broken femur, DCS acts as the external fixation and DCIR acts as the intra-medullary nail. Both are needed to effectively manage the complex patient.

#### Splenic trauma

Avoiding splenectomy in trauma is of particular importance as the majority of trauma patients are of a younger cohort and thus avoiding lifelong monitoring and antibiotic cover is preferred whenever possible. The role of IR in splenic trauma has been well documented, [23] albeit with some ccontroversy that remains over which subset of patients require DCS, or NOM ( which includes IR).

The SQUIRTS study [24] have retrospectively evaluated a protocol for routine splenic artery embolization (SAE) for all high-grade spleen injuries in a single trauma center, with 570 patients in 10 years. The authors confirmed that high-grade splenic injuries (III-V) undergoing splenic artery embolization (SAE) have favourable outcomes in comparison to off- protocol standard-of-care management. The main learning point is that patient pathways for high-grade splenic injury should include IR and routine embolization.

Breeding et al. also demonstrated good outcomes in high-grade splenic injuries undergoing embolization [25].

The application of DCIR in splenic trauma focuses on the earlier mentioned mantra of be as selective as time allows. Proximal splenic artery embolization (pSAE) is a quick, low risk and effective method of managing splenic injury. The aim of pSAE is to reduce the perfusion pressure enough to allow the bleeding to stop without causing splenic infarction but allowing the splenic parenchyma to heal. This technique is particularly effective in patients with higher grade injury with multifoci pseudoaneurysms [26]. The benefits of pSAE is, lower radiation dose, reduced procedure time, reduced risk of end artery ischaemia and necrosis. The con of pSAE is that once the vessel has been occluded, re-intervention can be challenging and at times impossible.

Distal SAE (dSAE) is targeted, where the abnormal vessel is embolized which is normally a vessel that is bleeding or a pseudoaneurysm. The authors feel dSAE is more effective for patients with active extravasation of contrast or penetrating injury where the capsule which would create a tamponade effect has been compromised. Of note dSAE is a longer procedure and presents higher radiation dose. It is also believed that dSAE has a higher chance of infarction of the embolized segment, and higher complication rates, yet few studies have shown that technical and clinical complications are similar between the two groups [27–29]. Lin and colleagues also demonstrated no difference in outcome or mortality in pSAE versus dSAE [30]. In addition long-term splenic function is thought to be preserved in pSAE which further encourages its use. In cases of re-bleed, as the main splenic artery remains patient, re-intervention is possible and in theory there is a lower risk of re-bleed as the bleeding artery has been occluded [30]. DCIR in splenic artery embolization includes both pSAE and dSAE and as such it is the patient’s clinical status and anatomy that dictates which technique is performed.

### Resuscitative Endovascular Balloon Occlusion of the Aorta (REBOA)

Another remarkable and controversial application of DCIR is REBOA, which emerged as a promising procedure in emergencies relatively recently [31]. It is a minimally invasive endovascular intervention aimed at controlling bleeding while maintaining cerebral and cardiac perfusion in cases of major haemorrhage. The basis of REBOA is to control non-compressible torso-abdominal hemorrhages. It allows the trauma team time to resuscitate the patient while they are transferred to CT, IR or the operating theatre.

The balloon can be inflated in three aortic zones, depending on the location of bleeding: Zone 1 extends from the left subclavian artery to the celiac artery, Zone 2 extends from the celiac artery to the lower renal artery. Zone 3 extends from the lower renal artery to the aortic bifurcation [32]. The function of REBOA is to occlude the aorta proximal to the presumed bleeding site in the abdomen, pelvis, or both, replacing traditional surgical aortic cross-clamping (ACC).

Besides theoretical advantages, the efficacy of REBOA is a matter of controversy. Some studies have indicated that REBOA is more effective than ACC in terms of prognosis and reducing surgical morbidity [33–35], while others have suggested that it is associated with a poorer prognosis [36–38]. The UK-REBOA trial has indicated that REBOA increases the risk of death and prolongs the time to definitive hemostasis in comparison to standard-of-care treatment for hemodynamically unstable trauma patients [36]. Martinez Hernandez et al. performed an analysis of this trial, concluding that it cannot be stated that REBOA increases mortality compared with standard care alone in trauma patients with exsanguinating hemorrhage due to a few limitations of the study like randomization of patients, sample size, device used and experience of the operators [39].

A Japanese descriptive analysis by Hoshi et al. investigated the use of REBOA over 18 years and suggested that for patients with similar disease severity, the use of REBOA may have contributed to a reduction in mortality over time. Another noteworthy observation from this study is the gradual and steady increase in the number of centers where this procedure is conducted as well as the number of procedures executed. This may be attributed to the growing recognition of the procedure [40].

Although the use of REBOA has been mostly evaluated to manage trauma patients, it is increasingly being used also for other types of bleeding, such as vascular emergencies, peri-partum hemorrhages (PPH) [41], and gastrointestinal and iatrogenic or spontaneous bleeding [32].

#### Peri-partum/post-partum haemorrhage

Post-partum hemorrhage is responsible for approximately 70 000 deaths per year [42]. Complications such as placental implantation anomalies and uterine atony post-delivery are responsible for primary post-partum hemorrhage [43]. Historically these patients were subjected to hysterectomy post-delivery (vaginal or Caesarian section), however balloon occlusion of internal iliac arteries and, if needed, subsequent uterine artery embolization has offered a new lifeline for these patients. In severe hemorrhage, hemodynamic control can be achieved by inserting a balloon catheter into the internal iliac artery (IIA), common iliac artery, or aorta (REBOA) [44].

We acknowledge the controversy and challenges of the latter option and advice to avoid this when possible. To avoid PPH, temporary balloon occlusion is utilized as a prophylactic therapy for bleeding in aberrant placenta implantation or those with history of previous PPH or at risk of bleeding. Prophylactic balloon occlusion involves inserting balloon catheters into the anterior divisions of IIAs bilaterally before a cesarean section. This can be performed under low dose fluoroscopy without the need for digital subtraction angiography. The confirmation of balloon position using fluoroscopy is sufficient. The balloon is then inflated after successful delivery of the baby and kept inflated until the bleeding has been controlled or uterine artery embolization is required. The balloons are removed either immediately after or the day after surgery.

Clinical success rates of up to 86% have been reported when using prophylactic balloon occlusion of the anterior division of IIA, transfusion rate, and reducing the rate of hysterectomies [45, 46].

Prophylactic balloon catheter occlusions and/or uterine artery embolization both play a crucial role in the management of peri-partum hemorrhage.

## Discussion

This review highlights the use of IR as an adjunct to the resuscitation of the trauma patient. Therefore it seems appropriate to think about changing the decision-making paradigm for trauma patients, to include IR as a minimally invasive procedure in resuscitation management and away from the NOM heading it currently sits under.

The absence of pre-established institutional protocols for multidisciplinary collaboration correlates with a delay in IR activation and, consequently, with poor outcomes [47–49]. International guidelines state clearly that IR should be on site as soon as possible. The American College of Surgeons Committee on Trauma (ACS-COT) recommends that the IR team should be available in level I and level II trauma hospitals within 30 min; the Society of Interventional Radiology (SIR) position statement for endovascular procedures in trauma, as well as the Cardiovascular Interventional Society of Europe (CIRSE), stated that the IR team should be prepared within 60 min [12, 13, 50].

Okada et al. reported a survival benefit generated by DCIR and DCS collaboration. According to their protocol, all indications for IR procedures or surgeries were discussed in a multidisciplinary fashion between emergency physicians, IR, and the surgical team. Both the IR and surgical teams were alerted by the pre-hospital emergency medical service regarding a patient in shock following a trauma. The IR team performed the procedures with support from the stand-by surgical team. Conversely, the surgical team performed the surgeries with the support of the stand-by IR team. If hemostasis could not be achieved within one hour, a switch to surgery or IR was considered. In their analysis patients were assigned to an embolization group (EG), a surgery group (SG), or a combination group (CG) according to their treatment and compared with the probability of survival (Ps) score calculated using the trauma and injury severity score (TRISS) methodology. In all three groups, the Ps scores were exceeded, in particular the survival rate in the CG was 15.5% higher than the calculated probability of survival [51].

Furthermore, a trauma hybrid operating room, in which both IR procedures and surgeries can be performed simultaneously, has been associated with earlier hemostasis, which in turn leads to fewer blood transfusions, infectious complications, and days on ventilator support [52, 53].

## Conclusions and future perspectives

The development of technologies and knowledge has elevated IR to be a fundamental pillar in the management of bleeding patients, thus deserving to take a seat at the table of the trauma team. Establishing a team training for DCIR in resuscitation practices is vital to the conception of IR as an arm of DCS and not as non-operative or ‘conservative’ management. The role of DCS is established and still a vital step in the management and good outcomes, however collaborative work between, emergency physicians, surgeons and IR is the only way we can ensure the full complement of treatment options is available to trauma and emergency patients to ensure the best outcomes.

Future perspectives for DCIR include an improved team workflow, with early activation of the interventional radiology service that can take part in the decision-making process as a clinician and not only as an operator.

## Data Availability

All content is what is available.

## Abbreviations

IR: Interventional radiology
DCS: Damage control surgery
DCIR: Damage Control IR
REBOA: Resuscitative Endovascular Balloon Occlusion of the Aorta
NOM: Non operative management
ABCDE: Airway, breathing, circulation, disability, and environment
WSES: World Society of Emergency Surgery
DSI: Damage Surgery Intervention
CT: Computed tomography
CEIR: Convention emergency interventional radiology
NSE: Non-selective embolization
PPP: Pre-peritoneal pelvic packing
SAE: Splenic artery embolization
pSAE: Proximal splenic artery embolization
dSAE: Distal SAE
ACC: Aortic cross-clamping
PPH: Peri-partum hemorrhages
ACS-COT: American College of Surgeons Committee on Trauma
SIR: Society of Interventional Radiology
CIRSE: Cardiovascular Interventional Society of Europe
EG: Embolization group
SG: Surgery group
CG: Combination group
Ps: Probability of survival
TRISS: Trauma and injury severity score
